# Therapeutic Targeting of the IGF Axis

**DOI:** 10.3390/cells8080895

**Published:** 2019-08-14

**Authors:** Eliot Osher, Valentine M. Macaulay

**Affiliations:** Department of Oncology, University of Oxford, Oxford, OX3 7DQ, UK

**Keywords:** IGF, type 1 IGF receptor, IGF-1R, cancer, acromegaly, ophthalmopathy, IGF inhibitor

## Abstract

The insulin like growth factor (IGF) axis plays a fundamental role in normal growth and development, and when deregulated makes an important contribution to disease. Here, we review the functions mediated by ligand-induced IGF axis activation, and discuss the evidence for the involvement of IGF signaling in the pathogenesis of cancer, endocrine disorders including acromegaly, diabetes and thyroid eye disease, skin diseases such as acne and psoriasis, and the frailty that accompanies aging. We discuss the use of IGF axis inhibitors, focusing on the different approaches that have been taken to develop effective and tolerable ways to block this important signaling pathway. We outline the advantages and disadvantages of each approach, and discuss progress in evaluating these agents, including factors that contributed to the failure of many of these novel therapeutics in early phase cancer trials. Finally, we summarize grounds for cautious optimism for ongoing and future studies of IGF blockade in cancer and non-malignant disorders including thyroid eye disease and aging.

## 1. Introduction

Insulin like growth factors (IGFs) are small (~7.5 kDa) ligands that play a critical role in many biological processes including proliferation and protection from apoptosis and normal somatic growth and development [1]. IGFs are members of a ligand family that includes insulin, a dipeptide comprised of A and B chains linked via two disulfide bonds, with a third disulfide linkage within the A chain. The two IGF ligands, IGFs–1 and 2, display 67% identity to each other and a high degree (~45–52%) of sequence homology with the A and B chains of insulin, but differ due to retention of the bridging C-domain, and a C-terminal D-domain extension [2]. Like insulin, the IGFs have three internal disulfides that maintain correct folding and permit canonical functions. The functions of IGF-1, IGF-2 and insulin are mediated through association with the cell surface receptor tyrosine kinases (RTKs) type 1 IGF receptor (IGF-1R), and insulin receptor (INSR) [3]. 

The high degree of homology between IGF-1R and INSR was apparent from the initial determination of the IGF-1R primary structure [4]. IGF-1R is a ~440–kda heterotetrameric α_2_β_2_ receptor with ~50% sequence homology to INSR, that is bound with high affinity by IGF-1 (~1–5 nM) and with significantly lower affinity (4–5 fold and >100-fold lower, respectively) by IGF-2 and insulin [5,6,7]. The two alpha subunits are extracellular and together comprise the binding site for a single molecule of ligand, whereas the two beta subunits are transmembrane structures that include the intracellular tyrosine kinase domains [3,8]. Ligand binding induces a conformational change that activates the β subunit kinase domain resulting in autophosphorylation of specific tyrosine residues, which appears to be the critical step in receptor activation [9] This in turn leads to recruitment and phosphorylation of the docking proteins insulin receptor substrates (IRS-1/2) and Shc, ultimately resulting in the activation of multiple signaling pathways, of which the two most well-characterized are Phosphoinositide 3-kinase-Protein kinase B (PI3K-AKT) and RAS-Mitogen-activated protein kinase (MAPK) [10,11]. These pathways are also activated by other RTKs, including Epidermal growth factor receptor (EGFR), Fibroblast growth factor receptor (FGFR) and Hepatocyte growth factor receptor (HGFR, MET); the IGF axis is known to engage in complex cross-talk with these pathways, and also with steroid hormone nuclear receptors, cell-matrix and cell-cell adhesion signaling components [12,13].

Despite the high degree of structural homology between IGF-1R and INSR, and the fact that the receptors signal via many common mediators, the two receptor signaling axes have undergone considerable functional divergence. As will be discussed below, this close structural relationship has posed a significant problem for development of agents to block the IGF axis. The primary role of INSR is anabolic control and glucose homeostasis; the classical receptor isoform responsible for these functions is known as INSR-B [14]. INSR-B is largely expressed in adult differentiated cells, notably the key insulin target tissues liver, muscle and adipose tissue, is encoded by 22 exons (11 exons encoding the alpha subunit and 11 the beta subunit), and binds insulin with high affinity but has a very low affinity for IGFs [15]. There is also a variant isoform, INSR-A, which is encoded by 21 exons due to exon 11 skipping, resulting in the absence of a 12 amino acid sequence at the α subunit carboxyl terminus [16]. This isoform is expressed by fetal tissues and cancers, and is capable of being activated by insulin and IGF-2 [17,18]. Cells that express both IGF-1Rs and INSRs express hybrid receptors formed as heterodimers between IGF-1R and INSR-A or B, with ligand binding characteristics and functions that are not fully characterized [19]. Unlike insulin that is synthesized and stored in the β islet cells of the pancreas and secreted in response to blood glucose, IGFs are expressed by multiple cell types and not stored prior to secretion [20]. Most circulating IGF-1/2 is secreted by the liver, IGF-1 secretion being strongly and IGF-2 weakly regulated by growth hormone (GH); indeed when first identified, IGFs were termed somatomedins, reflecting their role as GH mediators [21,22]. 

There are several intrinsic mechanisms in place to regulate IGF bioactivity. Firstly, the majority of circulating IGF-1/2 is present in high affinity, with a calculated dissociation constant (K_D_) < 1 nM in inactive complexes with IGF binding proteins (IGFBPs) [23]. IGFBPs therefore prolong the circulating half-life of IGFs, although they can undergo proteolytic cleavage to release free IGFs [24]. It is increasingly recognized that IGFBPs have more complex functions that can promote IGF bioactivity, and they also have IGF-independent functions, as reviewed by [25,26]. Secondly, the expression of IGF-2 is regulated by genomic imprinting, being expressed in normal tissues only from the paternal allele [27]. Thirdly, there is a type 2 IGF receptor (IGF-2R) that is structurally distinct from IGF-1R and INSR, being a monomeric transmembrane protein that acts as a scavenger for circulating IGF-2 [28].

The importance of the IGF axis in normal growth has been comprehensively studied using mouse genetics [29]. *Igfr1* null mice display growth retardation, (60% of normal birth weight,) a high rate of neonatal death due to organ hypoplasia, and persisting growth retardation (~30% of wild-type weight) for those individuals surviving into adulthood [29,30]. In humans, excess GH production results in abnormally high circulating IGF-1 levels and gigantism or acromegaly, while subnormal IGF-1 levels due to GH deficiency cause dwarfism [31,32]. Laron syndrome, a rare form of dwarfism caused by GH resistance resulting from GH receptor mutation, was recognized in the late 1950s by pediatric endocrinologist Zvi Laron, and has been particularly informative for understanding the contribution of the IGF axis to cancer risk [33,34], as will be discussed below. Clinical studies have identified severe growth delay and mental retardation in individuals harboring complete or partial *IGF1* or *IGF1R* gene deletion or point mutation, such as *IGF1* V44M that results in ~90-fold reduced affinity for IGF-1R [35,36,37]. This review will discuss the importance of the IGF axis in human disease with an emphasis on the importance of IGF-induced IGF-1R activation, and will focus on the approaches that have been taken to inhibit this key protein-protein interaction.

## 2. Disease States Characterized by IGF Axis Activation

### 2.1. Cancer

Due to the ability of IGFs to bind potently to IGF-1R and activate pathways associated with cellular proliferation, the IGF: IGF-1R interaction has long been recognized for its contribution to cancer growth and propensity for metastasis [38,39]. Under normal physiological conditions IGF signaling is tightly regulated, as outlined above [23,25,28]. However, genetic abnormalities and/or chromosomal alterations can result in deregulated expression of IGF ligands and IGF-1R [40]. These changes can occur as primary driver events that predispose to malignancy. Examples include *IGF1R* gene amplification and mutation in other IGF axis genes, detected in breast cancer, gastrointestinal stromal tumor (GIST) and osteosarcoma [41,42,43]. Activating point mutations in *IGF1R* itself have not been reported, but there are reports of mutational inactivation or loss of heterozygosity of the anti-proliferative *IGF2R* in prostate cancer and uveal melanoma [44,45]. Loss of *IGF2* imprinting has been shown to drive development of malignancy in mouse models and is associated clinically with colorectal cancer, Wilms tumor and hepatocellular carcinoma [27,46,47,48,49].

It must be acknowledged that in the majority of common solid tumors, IGF axis deregulation is not itself the driver but occurs secondary to another molecular event that influences the expression of the ligands and/or receptors. As will be seen, this lack of a driver role is an issue for clinical use of drugs that block the IGF axis, particularly when used as monotherapy. The upregulation of IGF-1R that occurs frequently in common solid tumors is often secondary to loss of the negatively regulatory influence of tumor suppressor genes including *BRCA1*, *WT1*, *TP53* and *vHL* [50]. Even if not driver events, overexpression and/or activation of IGF axis components promotes canonical signaling via effectors including AKT and ERKs that contribute to resistance to cancer therapies including chemotherapy, radiotherapy, endocrine therapy and targeted agents [51,52,53,54,55,56]. It is increasingly recognized that tumor growth, metastasis and therapy resistance can be promoted by IGFs secreted by cellular components of the tumor stroma [57,58,59].

Recent studies have identified an IGF-inducible non-canonical function of IGF-1R: following internalization and clathrin-dependent endocytosis, the receptor is capable of translocating to the nucleus and acting as a transcription factor by binding to regulatory regions of DNA [60,61,62]. Our group has reported that nuclear IGF-1R is detectable in pre-invasive lesions and several types of invasive malignancy including prostate, renal and breast cancers, and is associated with adverse prognosis in renal cancer and advanced tumor stage in prostate cancer [61,63]. Furthermore, we showed that IGF-1R recruitment to the *JUN* and *FAM21A* promoters contributes to expression of these genes that mediate cell survival and motility, angiogenesis and chemo-resistance [63,64,65].

As well as promoting tumorigenesis and treatment resistance, IGFs contribute to the risk of developing cancer. This has been shown most clearly by the almost complete protection from cancer in patients with Laron dwarfism, who have very low levels of serum IGF-1 due to GH receptor mutation [33,34,66]. In addition to regulation by GH, circulating IGF-1 levels are known to be influenced by dietary factors including dairy and total protein intake [67,68,69]. It is now well-established that subjects in the general population who have serum IGF-1 levels at the upper end of the normal range are at increased risk of developing several types of cancer including prostate, breast and colorectal cancer [70,71,72,73].

### 2.2. Endocrine Disorders

#### 2.2.1. Acromegaly

Subjects with acromegaly, which is due to excessive GH secretion, have elevated levels of serum IGF-1 and are well-recognized to be at increased risk of colorectal cancer, with possible association also with breast, thyroid and prostate cancers and a recent report of multiple additional tumors including cancers of the lung, kidney, adrenal and GIST [74,75]. There are additional health risks associated with chronic exposure to high circulating IGF-1, including increasing bone fragility. This seems paradoxical given that IGFs are required for normal bone development [76]. However, excess IGF-1 secretion has been shown to compromise bone integrity and microstructure, leading to increased risk of vertebral fracture [77]. IGFs are also important for the growth and survival of cardiomyocytes, and untreated acromegaly can cause hypertrophic cardiomyopathy leading to cardiac failure [78].

#### 2.2.2. Diabetes

Several large longitudinal studies have investigated links between serum IGF-1 and risk of type 2 diabetes mellitus (T2DM), and have found evidence for an association between increased incidence of insulin resistance and T2DM in subjects with either low-normal or high-normal IGF-1 levels [79,80]. The factors contributing to this U-shaped association are incompletely understood, but may be due to the fact that IGF bioactivity is influenced not only by serum IGF-1 but also other IGF components including IGFBPs. The association with low IGF-1 is probably related to the insulin-like actions of IGF-1 that promote hypoglycemia, and the fact that IGF-1 suppresses secretion of GH, which itself causes insulin resistance [81,82]. Recombinant human IGF-1 (rhIGF-1) has been evaluated as treatment for diabetes, with evidence of improved glycemic control but significant adverse effects including worsening diabetic retinopathy [83,84]. Currently there are no indications for use of rhIGF-1 in diabetes treatment, but there is ongoing interest in exploiting IGF-dependent and -independent actions of IGFBPs, especially IGFBP -1 and -2, to influence insulin sensitivity [82].

#### 2.2.3. Thyroid Eye Disease

Graves’ disease (GD) is an autoimmune disorder caused by pathogenic thyrotropin (thyroid stimulating hormone, TSH) receptor autoantibodies (TRAb), which have agonist TSH-like actions leading to goitre and thyrotoxicosis [85]. Between 25 and 50% of patients with autoimmune thyroid disease develop thyroid-associated ophthalmopathy (TAO), with proptosis due to intra-orbital expansion of connective and fatty tissue [86]. Thyrotropin receptors are expressed by monocyte-derived orbital fibrocytes, which are induced by TRAbs to secrete glycosaminoglycans including hyaluronan, and pro-inflammatory cytokines including IL-16, TNF-alpha and Chemokine C-C motif ligand 5 (CCL5, RANTES) [87]. The involvement of the IGF axis in orbital tissue expansion is now well-established: IGF-1R is over-expressed by orbital fibroblasts and T and B cells of GD patients, and IGF-1 is known to synergize with thyrotropin and to regulate immune functions [88,89]. Furthermore, complexes containing thyrotropin, IGF-1R and INSR have been detected in orbital fibroblasts and thyroid epithelial cells, the IGF axis is required to maintain thyrotropin signaling, and anti-IGF-1R autoantibodies that activate IGF-1R are detectable in patients with GD [87,90,91]. Thus, although cross-talk between thyrotropin and IGF signaling has been recognized for over 30 years, it is only recently that IGF-1R activation has been implicated in driving the immunopathogenesis of GD [87,88,92,93].

### 2.3. Skin Diseases

#### 2.3.1. Psoriasis

IGF-1 appears to contribute to the epidermal hyperproliferation that characterises psoriasis. IGF-1Rs are upregulated in psoriatic lesions and are expressed by proliferating basal and suprabasal keratinocytes, likely accounting for the greater proliferative response to IGF-1 of keratinocytes from psoriatic skin compared with normal keratinocytes [94,95]. Reduction in IGFBP-3 expression in the epidermal rete pegs probably increases local IGF bioavailability, also contributes to increased proliferation [96,97]. 

#### 2.3.2. Acne 

A link between IGF-1 and acne is suggested by the known cross-talk between IGFs and androgens, which are implicated in acne pathogenesis [98,99], and by absence of acne in Laron dwarfs and the appearance of acne upon IGF-1 treatment [100]. In the general population, adults with acne are reported to have elevated levels of serum IGF-1, and IGF-1 levels correlate with acne severity [101,102]. The mechanism of this apparently causative association has been studied in preclinical models, where IGF-induced PI3K-AKT activation upregulates sterol-response element binding protein-1 to increase sebaceous lipogenesis, and also upregulates inflammatory cytokines [101,103,104].

### 2.4. Frailty and Lifespan

IGFs are important for maintenance of bone and muscle mass, and IGF-1 serum levels have been reported to be lower in the frail elderly compared with more robust subjects [105,106]. Paradoxically, the IGF axis is the target of inactivating mutations in long-lived model organisms including Caenorhabditis elegans, Drosophila melanogaster and mice [107,108]. Supporting the clinical relevance of these findings, *IGF1R* mutations in humans that attenuate IGF response have been found to be associated with longevity and a reduction in frailty [109]. Thus, there is a conflict in the literature between findings that IGFs promote muscle strength and bone density, and reports associating low IGF-1 bioactivity with reduced frailty. Potentially resolving this conflict, preclinical and population-based studies indicate that a high protein diet and high IGF-1 in middle age associate with increased cancer incidence and mortality, and promote tumour growth in mouse models, while a low protein diet and low IGF-1 are detrimental in old age [110]. 

## 3. Therapeutic Strategies for Targeting IGF-1R in Cancer

Given the functions of IGF-1R and the pathogenic associations of high IGF bioactivity, the IGF axis has been acknowledged as a target for therapeutic intervention. In fact, IGF-1R was the first RTK to be targeted by an inhibitory antibody in preclinical studies, when neutralizing antibody αIR3 was shown to inhibit growth of breast cancer cells in vitro and as xenografts in immunodeficient mice [111]. However, clinical evaluation of this approach was delayed by legitimate concerns about the potential for toxicity, due to inhibition of normal tissue IGF-1R and the co-inhibition of INSR [112,113]. From 2004–2005 onwards, a number of different strategies were developed for evaluation in the clinic. The principal strategies include molecular approaches, anti-IGF-1R antibodies, small molecule tyrosine kinase inhibitors (TKIs), IGF-1/2 neutralizing antibodies and IGF ligand TRAPs. Several reviews have addressed the important issues of clinical efficacy, the need for predictive biomarkers and selection of targets for co-inhibition e.g., [56,114,115,116,117]. We focus here on the physical properties of each class of agent and summaries their current clinical status.

### 3.1. Molecular Approaches

Prior to availability of Pharma IGF-1R inhibitory drugs, nucleic acid-based approaches were developed to target IGF-1R. The sequence specificity of these approaches generated tools that targeted IGF-1R specifically, reflecting concerns relating to INSR co-inhibition; these agents proved useful in proof of concept preclinical experiments, and some reached the clinic.

#### 3.1.1. Antisense Oligonucleotides 

Antisense-mediated downregulation of Igf-1 or Igf-1r was shown to inhibit growth of murine tumors in vivo, and induce an immune response that suppressed growth of unmodified tumors in syngeneic models [118,119]. These data encouraged clinical testing of IGF-1R antisense oligonucleotides (ASOs) in a pilot study in patients with malignant astrocytoma. IGF-1R ASOs were transfected ex vivo into autologous tumor cells that were re-implanted into the subcutaneous tissues, with response in 8/12 patients [120]. IGF-1R ASO modified to increase stability was shown to reduce epidermal hyperproliferation in human psoriatic skin xenografts in vivo [121]. This ASO was developed as a drug, ATL1101, that was shown to inhibit growth of prostate cancer xenografts following intraperitoneal administration [122]. ATL1101 was formulated into a cream and tested in a proof of concept clinical study in patients with psoriasis, reporting evidence of clinical benefit (https://www.sec.gov/Archives/edgar/vprr/0501/05012077.pdf), but has not apparently progressed further. 

#### 3.1.2. siRNAs

Soon after the fortuitous discovery of RNA interference (RNAi) in 1998, the technology was considered for therapeutic use [123]. RNAi is mediated by siRNAs, short (20–25 bp) double-stranded RNAs that recruit the RNA-silencing complex to silence target genes with high specificity via mRNA degradation, and can also induce epigenetic modification and transcriptional repression by interaction with the transcriptional machinery [124,125]. Our group reported that IGF-1R siRNA efficacy is influenced by secondary structure in *Igf1r* mRNA, leading to design of IGF-1R siRNAs that induced profound *IGF1R* silencing and enhanced tumor cell radiosensitivity [126]. Subsequently, we and others showed that *IGF1R* gene silencing enhances sensitivity to chemotherapy, ionizing radiation and targeted agents in prostate, renal, and esophageal cancer and HCC models in vitro and in vivo [127,128,129,130]. Major issues for clinical use include siRNA delivery and stability in vivo, although stabilized siRNAs can be effective following in vivo administration [131]. Currently 62 siRNA trials are registered on https://clinicaltrials.gov, although this approach has not been pursued for IGF-1R.

#### 3.1.3. Dominant Negative Receptors

Another nucleic acid-based approach to blocking IGF signaling was designed to exploit the knowledge that IGF-1R is a disulphide-bonded heterotetramer in which one molecule of ligand binds into a pocket formed of two IGF-1R alpha subunits [3,8]. Thus, the function of endogenous IGF-1R can be prevented by expression of dominant-negative receptor that complexes with a wild-type half receptor to allow ligand binding while lacking kinase activity. Dominant negative IGF-1Rs (dnIGF-1Rs) have been generated by expression of IGF-1R residues 1–486, encoding soluble receptor or IGF-1R 1–950 that is expressed at the cell surface [132,133,134,135,136,137]; these dnIGF-1Rs are capable of inducing apoptosis *in vitro* and inhibiting tumorigenesis and metastasis *in vivo*. Comparable dominant negative IGF-1R blockade has also been achieved using a mutant ‘decoy’ IGF-1 defective in integrin binding, which was shown to inhibit anchorage-independent growth *in vitro* and tumorigenesis *in vivo* [138]. Clinical application of the dominant negative approach is limited by technical issues related to delivery and duration of expression, and regulatory and safety considerations [139]. However, on a positive note, the preclinical efficacy of dnIGF-1Rs encouraged development of a related approach, the IGF-Trap, based on protein therapeutics (see below).

### 3.2. Anti-IGF-1R Agents

#### 3.2.1. IGF-1R Antibodies

**Figure 1:**
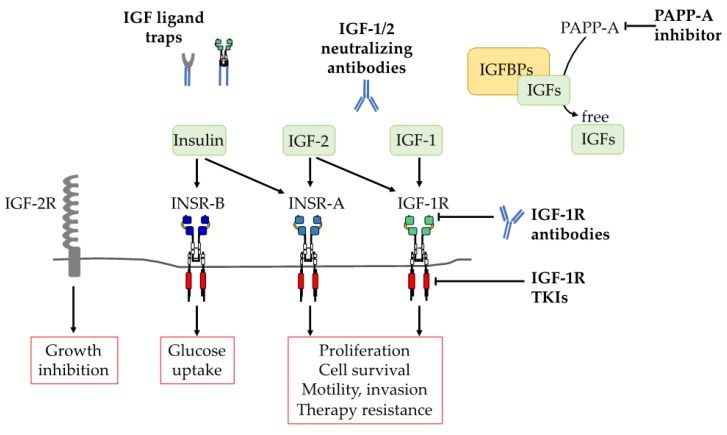
Overview of therapeutic strategies developed to inhibit the insulin like growth factor (IGF) axis. Figure shows the major components of the IGF axis and approaches to targeting by different classes of inhibitor. Information taken from references cited in the text.

The first major Pharma agents to be tested clinically were monoclonal antibodies (mABs) that bind to the IGF-1R alpha subunit, blocking IGF binding (Figure 1). This was a logical initial approach, given concerns over the risk of side-effects due to INSR co-inhibition [112]. Indeed, these antibodies exhibit exquisite specificity for IGF-1R over INSR, as summarized in Table 1 for the eight mABs that have been evaluated in early phase trials in cancer patients. Mechanism-based studies revealed that in addition to blocking IGF ligand binding and hence IGF-induced signaling, IGF-1R antibodies induce IGF-1R internalization and degradation [140]. This property has implications for toxicity: despite negligible affinity for INSR, some mABs bind IGF1R:INSR-A/B hybrid receptors, inducing their internalization and downregulation [141,142]. This is one likely cause for dose-limiting hyperglycemia in patients treated with IGF-1R antibody; other causes include the endocrine response to IGF-1R blockade, inducing hepatic secretion of GH and IGFBPs which can impair glucose tolerance [143]. Conversely, for those antibodies that do not cause INSR co-downregulation, compensatory signaling can be generated by activation of INSR-A or -B by insulin or IGF-2 [144,145,146,147,148,149], or by crosstalk with other RTKs including EGF and MET [150,151].

Despite early excitement generated by the activity of IGF-1R antibodies e.g., [153,157], all the IGF-1R antibody programs have been terminated by the Pharma companies that conducted the initial development and clinical trials. Some of the trials generated evidence of activity in patient subgroups, for example in patients with high free circulating IGF-1, and those whose tumors harbored mutant KRAS [114,161,177]. However, there was little or no cross-talk between programs, so for example Dalotuzumab was tested in KRAS wild-type colorectal cancer, with no evidence of activity [167]. Patients with Ewing sarcoma have shown evidence of benefit in a number of trials [157,162,170,173]; overall, responses have been seen in ~10% of Ewing patients [178]. Two drugs remain in active development. Firstly, Ganitumab was acquired from Amgen by Dr Patrick Soon-Shiong, chairman and CEO of NantCell. This antibody is currently being tested in combination with SRC inhibitor dasatinib in patients with rhabdomyosarcoma (NCT03041701), and with chemotherapy in Ewing sarcoma (NCT02306161), for which Ganitumab has been granted orphan drug status by the FDA. Secondly, Teprotumumab (R1507) is showing promising results in TAO, as discussed in Section 4.3.

#### 3.2.2. Tyrosine Kinase Inhibitors (TKIs)

Small molecule IGF-1R TKIs were developed by Pharma to block IGF-1R kinase activity and hence suppress IGF signaling. Whilst many experimental IGF-1R TKIs have shown preclinical efficacy e.g., [179], few have undergone clinical evaluation (Table 2), and results have been disappointing. Because of high levels of sequence homology (~85%) between IGF-1R and INSR-A/B kinase domains, including 100% identity in the ATP-binding cleft [180], the ATP-competitive TKIs also inhibit INSR [181,182]. This can be advantageous in blocking compensatory INSR-A signaling [145] but also problematic in compromising metabolic insulin signaling via INSR-B, leading to hyperinsulinemia and dose-limiting hyperglycemia [183]. Where managed by delays and dose reductions, this and other toxicities resulted in reduced exposure to co-treatments e.g., erlotinib [184]. Furthermore, the relatively short half-life of small molecule TKIs could have resulted in only intermittent blockade of target receptors. Indeed, given understandable caution around the potential for toxicity, the linsitinib trials included formal evaluation of intermittent dosing [185,186,187]. It is plausible that high circulating IGF and insulin levels, induced as endocrine feedback during periods of receptor blockade, could have driven rebound tumor growth when receptors were responsive. Supporting this possibility, some patients treated with linsitinib did experience hypoglycemia [185,186]. 

Non-ATP competitive IGF-1R inhibitors AXL1717 and INSM-18 inhibit IGF-1R without INSR blockade, and also inhibit additional less closely related targets [188,189,190]; see Table 2. The inhibition of multiple cancer targets could be advantageous, but these agents have had limited clinical success, and programs for XL-228 and INSM-18 have been discontinued. In a Phase I trial, AXL1717 induced responses in 4/9 (44%) of patients with relapsed malignant astrocytomas [191], and has been granted orphan drug designation for this indication. Further clinical trials are planned using a new formulation (www.axelar.se/news/FDA-Grants-Orphan-Drug-Designation-for-AXL1717-for-the-Treatment-of-Glioma).

### 3.3. Targeting IGF Ligands

#### 3.3.1. IGF Neutralizating Antibodies

The major issues associated with IGF-1R inhibition led to identification of an alternative therapeutic strategy that specifically targets the IGF ligands (Figure 1). Two Pharma companies generated IGF neutralizing antibodies that have entered clinical trials. Dusigitumab (MEDI-573, Medimmune) is a fully human IgG2λ monoclonal antibody with picomolar binding affinity for human IGF-1 and IGF-2 (KD 294 and 2 pmol/L respectively) that inhibits IGF-1 and IGF-2 -induced IGF-1R phosphorylation with IC_50_ concentrations of 0.97 and 0.2 µg/mL, respectively. Thus, dusigitumab blocks IGF-induced IGF-1R and IR-A activation but has no detectable binding to insulin [200]. In a Phase I trial, dusigitumab was well-tolerated although without evidence of single agent activity [201]. A Phase Ib/II trial was conducted in patients with hormone receptor positive (HR+) metastatic breast cancer in combination with aromatase inhibitor, but there was no significant difference in progression-free survival vs. aromatase inhibitor alone (NCT01446159). Following the acquisition of Medimmune by AstraZeneca, it has been reported that the dusigitumab program is to be discontinued on the conclusion of this trial (https://labiotech.eu/medical/axed-checkpoint-inhibitor-astrazeneca/).

Boehringer Ingelheim also generated an IGF neutralizing antibody, xentuzumab (BI 836845), that blocks IGF-induced IGF-1R and INSR-A functions. This humanized IgG1 monoclonal antibody binds IGF-1 and IGF-2 with high affinity (0.07 and 0.8 nmol/L, respectively), with IC_50_ for inhibition of IGF-1R phosphorylation in response to IGF-1 of 0.6 nmol/L and IGF-2 of 7.5 nmol/L [202]. Unlike MEDI-573, xentuzumab cross-reacts with murine IGFs, allowing preclinical in vivo assessment and revealing growth inhibition in rats, and anticancer activity with rapamycin in human tumor xenografts [202,203]. Xentuzumab is being tested clinically with afatinib in EGFR-mutant lung cancer (NCT02191891), in prostate cancer with enzalutamide (NCT02204072) and in HR+ breast cancer with everolimus and exemestane (NCT02123823). In advanced prostate cancer, the addition of xentuzumab to enzalutamide did not improve outcomes overall, although there was evidence of PFS prolongation in patients with high tumor *IGF1* mRNA, albeit in a small sample [204]. In breast cancer, there was evidence of activity in patients with non-visceral disease [205]. Activity in patients with predominant bone metastases may reflect preclinical evidence that tumor cells are primed to metastasize to bone by high IGF-1 secreted by stromal components of the primary tumor, suggesting that bone metastases may reflect IGF dependency [76,206]. These results have prompted a further Phase II trial of xentuzumab in breast cancer patients with non-visceral disease (NCT03659136).

#### 3.3.2. IGF Ligand-TRAPs

Cell surface receptors can be targeted utilizing soluble traps that bind their ligands with high affinity, inhibiting activation of cognate receptors (Figure 1). Examples of this approach are the TNF-alpha inhibitor etanercept for treatment of rheumatoid arthritis and VEGF-TRAP aflibercept for cancer and retinal disease [207,208,209,210]. Two approaches have been taken to develop an IGF ligand trap for cancer therapy. To trap IGF-2, the ligand binding domain of IGF-2R was mutated and fused to IgG1 Fc domain to generate a homodimer capable of greatly enhanced high affinity IGF-2 binding [211,212]. Secondly, using a strategy evolved from the dnIGF-1R approach [134], an IGF-TRAP has been generated by fusing the IGF-1R extracellular domain to the Fc region of human IgG_1_. This agent binds potently to both IGF ligands and much more weakly to insulin, and suppresses growth of breast cancer xenografts and colon and lung cancer liver metastases in vivo [213]. One issue for this type of Fc-fusion protein is the formation of high molecular weight complexes due to the propensity of cysteine to form disulfides between Fc fragments. To address this, the first generation IGF-TRAP has since been modified by introduction of a flexible linker and cysteine-serine substitutions in the Fc hinge region, preventing oligomer formation; this improved agent has been shown to have anti-cancer activity in an experimental colon carcinoma metastasis model [214]. There are currently no IGF-TRAPS in clinical use, but the preclinical data are promising.

#### 3.3.3. Recombinant IGFBPs

Given their role as naturally occurring IGF inhibitors, IGF binding proteins have been identified as a focus for drug development. IGFBP3 is the principal circulating IGFBP, and also has IGF independent actions in the DNA damage response and EGF signaling; the latter effect is mediated by sphingosine kinase-1 (SphK1) and can be blocked by SphK1 inhibition [215,216]. Expression of recombinant human IGFBP3 (rhIGFBP3) has been explored to block IGF-dependent actions of IGFBP3, and was shown to have anticancer activity in vitro and in vivo [217]. This prompted development by Insmed of rhIGFBP3 as a protein therapeutic alongside the IGF-1R:HER2 inhibitor INSM-18, but development of both agents was subsequently discontinued.

#### 3.3.4. PAPP-A Inhibition

Pregnancy-associated plasma protein-A (PAPP-A) is a metalloprotease that enhances IGF bioactivity by proteolytic cleavage of IGFBPs, particularly IGFBP-4 [218]. Anti-PAPP-A antibody has been shown to have significant anti-cancer activity in vivo, inhibiting formation of malignant ascites in immunodeficient mice injected intraperitoneally with patient-derived ovarian cancer or ascites [219]. Reflecting the influence of IGF bioactivity on longevity, PAPP-A null mice have been found to have prolonged lifespan, significantly reduced incidence of fatal neoplasia and reduction in degenerative changes including cardiac and renal disease [220]. As a result, there is now major interest in targeting PAPP-A as an approach to block IGF bioactivity in aging research [221].

### 3.4. Natural Products That Inhibit the IGF Axis 

Whilst arguably the weakest of all the therapeutic classes, natural products have been known to have medicinal benefits for centuries and are the focus of interest for early stage drug discovery [222,223]. Many naturally-occurring organic compounds have been shown to block growth factor signaling, including blockade of the IGF axis [224]. Natural products reported to inhibit IGF actions include curcumin from turmeric, genistein from soy products, and apigenin and quercetin, present in fruits, vegetables and grains [225,226,227]. For example, curcumin has been shown to downregulate IGF-1R and INSR in colorectal cancer cells, and to block IGF-induced activation of IGF-1R, PI3K-AKT and mTOR, suppressing carcinogen-driven skin tumorigenesis in an Igf-1 driven model [228,229]. This is an active area of clinical research and many trials are evaluating these agents: currently ~200 for curcumin and ~60 for quercetin (https://clinicaltrials.gov).

## 4. Therapeutic Use of IGF Axis Inhibitors: Current Status 

### 4.1. Negative Trials of IGF-1R mABs and TKIs in Cancer Patients

Despite encouraging results in early clinical trials, IGF-1R inhibitors have not proved to have useful single agent activity in patients with cancer, with the possible exception of Ewing sarcoma [157,162,170,173,178]. Factors that may contribute to lack of efficacy include compensatory signaling via INSR-A, IGF-1R:INSR hybrid receptors and other RTKs including EGFR and MET [117,145,150,151]. To address this issue, trials are exploring effects of multiple targeted agents in combination [230,231]. An important consideration especially for relatively short-acting IGF-1R:INSR TKIs is the potential for rebound pathway activation that may result from high IGF and insulin levels induced during periods of receptor blockade, which are then available to activate IGF-1R and INSR signaling when the receptor blockade is released [185,186]. This regulation of ligand levels by endocrine feedback is a factor that differentiates the IGF: insulin axis from other RTK pathways, and is clearly relevant to therapy. Another factor that could have compromised outcomes in trials of IGF-1R inhibitors with chemotherapy is the possibility that cell cycle arrest due to IGF axis inhibition may protect from phase specific cytotoxic drugs [232,233], reviewed in [56]. As a result of the negative results in Phase II and III, most of the programs have been terminated. 

### 4.2. Potential Grounds for Cautious Optimism

Renato Baserga concluded his most recent review ‘The decline and fall of the IGF receptor’ with the words ‘*spes ultima dea*’ (hope is the last goddess) [234]. Several aspects do give grounds for cautious optimism. Firstly, single-agent activity has been observed in trials of patients with Ewing sarcoma [178], clinical evaluation of IGF-1R mAB Ganitumab is ongoing in patients with Ewing and rhabdomyosarcoma, and Ganitumab has been granted orphan drug status in Ewing sarcoma. Secondly, the development of IGF neutralizing antibodies provides a means of blocking IGF-1R and INSR-A without compromising insulin signaling via INSR-B [201,202], thereby avoiding dose-limiting hyperglycemia that contributed to adverse outcomes in IGF-1R inhibitor trials. Recent data from the Phase II trial of xentuzumab in ER+ breast cancer reported no significant hyperglycemia, and provided initial evidence of activity in patients with non-visceral metastases [205]. Finally, it is clear that many of the negative trials contained patients who experienced very durable, sometimes exceptional, responses, e.g., [157,235,236]. Much effort has gone into the search for predictive biomarkers to guide patient selection (reviewed in [56]). Although no useful biomarker has yet been identified, this information could yet be generated by preclinical research e.g., [237,238,239], and ongoing trials. Support for this statement comes from the preliminary evidence of benefit from xentuzumab in breast cancer patients with predominant bone metastases, and prostate cancer patients whose tumors express high *IGF1* mRNA [204,205].

### 4.3. Repurposing IGF Axis Inhibitors for Non-Malignant Disorders

Considering that many anti-cancer drugs have undergone laborious and costly development and clinical testing, it is an attractive prospect to consider their re-purposing for other disorders where the IGF axis plays a fundamental role. Two disease areas in particular are the focus of active investigation, to explore the potential of IGF-1R inhibition for non-cancer indications. Firstly, as described above (Section 2.2.3.), IGF axis activation has been recognized as making a major contribution to TAO, providing the impetus for clinical trials of IGF-1R blockade. In a preclinical study, re-purposed fully human anti-IGF-1R mAB Teprotumumab (R1507, Table 1) was shown to downregulate IGF-1R and thyrotropin receptor in fibrocytes and inhibit IGF- and TSH- dependent AKT phosphorylation and TSH-mediated IL-6 and IL-8 induction [240]. In a randomized Phase II trial (NCT01868997), Teprotumumab was evaluated in patients with active, moderate-to-severe TAO, with response in 29/42 (69%) of patients on the Teprotumumab arm compared with 9/45 on placebo (20%, *p* < 0.001). Evidence of benefit was apparent after only 6 weeks’ treatment, and Teprotumumab was well-tolerated apart from hyperglycemia in diabetic patients [241]. Outcomes of the subsequent Phase III OPTIC trial (NCT03298867) were reported in April 2019. Patients with TAO achieved significantly greater benefit from Teprotumumab compared with placebo, 82.9% vs. 9.5% (*p* < 0.001) achieving the primary end point of ≥2 mm reduction of proptosis (www.endocrinologyadvisor.com/home/conference-highlights/aace-2019/teprotumumab-effectively-reduces-proptosis-in-active-thyroid-eye-disease/). These data are extremely encouraging, and support introduction of Teprotumumab as standard of care in TAO [241,242]. Secondly, given data described above (Section 2.4.) demonstrating a role for IGFs in the regulation of healthy aging [107,108,109], there is interest in developing approaches to block IGF signaling for this indication [243]. Support for this idea has been recently provided by a report that a murinized version of Ganitumab improved the health and lifespan of female mice when administered for 6 months from 18 months of age, the mouse equivalent of mid-50s in humans [244]. 

## 5. Conclusions

This review has summarized the contribution of the IGF axis to malignant and non-malignant conditions, and the main strategies that have been or are being developed to block IGF signaling. The success of Teprotumumab in TAO is encouraging, and this seems likely to be the recipient of the first license for an anti-IGF-1R agent. IGF-1R inhibitors may also continue to be a focus of interest in in aging research. While compelling preclinical data supported development of IGF-1R targeted drugs as anti-cancer treatments, there have been no unequivocally positive trials. As a result, no IGF-1R mAB or TKI is licensed for use in patients with cancer. However, reports of exceptional responders to IGF axis blockade supports the concept of IGF targeting as cancer therapy. Mature data on the efficacy of IGF neutralizing antibody xentuzumab are awaited, and there is ongoing research to identify predictive biomarkers, which are essential for effective use of any targeted therapy. 

## Figures and Tables

**Table 1:** Anti- type 1 IGF receptor (anti-IGF-1R) antibodies evaluated in clinical trials in cancer patients.

Antibody	Humanized	Class	Potency (IC_50_) IGF-1R	Potency (IC_50_) INSR	Clinical Trial Phase	References
Figitumumab (CP-751,871)	Fully human	IgG2a	1.8 nM	N/A	III	[152,153,154,155,156]
Ganitumab (AMG 479)	Fully human	IgG1	2 nM	>50 nM	III	[157,158,159]
Teprotumumab (R1507)	Fully human	IgG1	0.5 nM	N/A	II	[160,161,162,163]
Dalotuzumab (MK-0646)	Humanized	IgG1	1 nM	N/A	III	[164,165,166,167]
Cixutumumab (IMC-A12)	Fully human	IgG1	0.6–1 nM	N/A	II	[168,169,170,171]
Robatumumab (SCH 717454)	Fully human	IgG1	2.7 nM	N/A	II	[172,173]
Istiratumab (MM-141)	Engineered human	IgG1 with 2 scFvs	2 nM	Bispecific IGF-1R/ErbB3	II	[174]
BIIB022	Fully human	IgG4	<10 nM	N/A	I	[175,176]

**Table 2:** IGF-1R Tyrosine Kinase Inhibitors (TKIs) that have undergone clinical testing.

Drug Name	Mode of Inhibition	Potency (IC_50_) IGF-1R INSR		Additional Targets	Clinical Trial Phase	References
Linsitinib (OSI-906)	ATP competitive	35 nM	75 nM	N/A	III	[145,181,184,185,186,187,192,193]
BMS-754807	ATP competitive,	1.8 nM	1.7 nM	MET, RON, TrkA/B, AurA/B	II	[182,194]
XL-228	ATP competitive	1.6 nM	N/A	BCR-ABL, AurA, SRC, LYN	I	[189,195]
AXL1717 (Picropodophyllin)	Non-ATP competitive	40 nM	N/A	Microtubules	II	[190,191,196,197]
Masoprocol (INSM-18, nordihydroguaiaretic acid)	Non-ATP competitive natural product of Larrea divaricata	31 µM	N/A	HER2	II	[188,198,199]
